# Brazilian front-of-package nutrition labeling: consumer perceptions on social media platform X

**DOI:** 10.3389/fnut.2025.1666794

**Published:** 2025-10-21

**Authors:** Mariana Ribeiro, Juliana de Paula Matos, Carolina Kikuta, Thais da Silva Marques, Beatriz Escaler Arruda, Paula Martins Horta, Ana Paula Bortoletto Martins, Laís Amaral Mais

**Affiliations:** ^1^Institute for Consumers Defense (Idec), São Paulo, Brazil; ^2^School of Public Health, University of São Paulo (FSP/USP), São Paulo, Brazil; ^3^Center for Epidemiological Research in Nutrition and Health (Nupens), University of São Paulo (USP), São Paulo, Brazil; ^4^Department of Nutrition, Federal University of Minas Gerais (UFMG), Belo Horizonte, Brazil; ^5^Department of Preventive Medicine, School of Medicine, University of São Paulo (USP), São Paulo, Brazil

**Keywords:** food labeling, front-of-pack nutrition labeling, social media, public understanding, Brazil, content analysis, policy evaluation

## Abstract

**Objective:**

This study aimed to analyze how the Brazilian front-of-package nutrition labeling (FOPNL) policy has been represented and discussed on the social media platform X (formerly Twitter).

**Methods:**

A cross-sectional observational study was conducted based on 2,323 posts published by personal user accounts on X between October 2020 and April 2024. All posts were written in Portuguese and contained terms related to Brazilian FOPNL. The posts analysis comprised: (i) publication type (science, information, opinion, regulation), (ii) positioning on FOPNL (positive, moderately positive, neutral, moderately negative, negative), and (iii) thematic content, based on eight thematic categories: nutrients of concern, health risk, regulatory aspects, no effect, support for the regulation, critiques and concerns, decision-making and others. Descriptive statistics were used to examine the distribution of posts over time and the relationship between thematic categories and positions on FOPNL.

**Results:**

The number of posts increased progressively over the study period. The most prevalent thematic categories were nutrients of concern (92.0% CI 95% 90.86–93.07), critiques and concerns (31.2% CI 95% 29.40–33.17) and no effect (29.8% CI 95% 28.00–31.73). Favorable perceptions on FOPNL were noted, particularly in posts supporting the regulation (67.36%), whereas negative positions were more commonly found in the “critiques and concerns” category (91.36%). Thematic analysis revealed a variety of user reactions, including expressions of surprise, discomfort, or dissatisfaction, often conveyed through ironic or sarcastic tones.

**Conclusion:**

The implementation of FOPNL in Brazil was accompanied by increasing public engagement over time. It was possible to understand consumers’ positions and sentiments related to FOPNL through the posts analysis on social media platform X, ranging from positive to negative. These findings highlight the importance of monitoring not only regulatory compliance but also public attitudes toward health policies, as a strategy to inform policy refinement, strengthen public trust, and enhance the impact of nutrition labeling initiatives.

## 1 Introduction

In recent decades, the global prevalence of chronic non-communicable diseases (NCDs) has increased significantly, with type 2 diabetes, hypertension, and obesity emerging as major public health concerns in Brazil (1). This trend is influenced by multiple factors, notably the growing consumption of ultra-processed food products (UPFP), which are becoming increasingly prominent in the Brazilian dietary pattern (2). A substantial body of evidence has associated UPFP consumption with an elevated risk of adverse health outcomes, including NCDs (3).

Front-of-package nutrition labeling (FOPNL) is a public health strategy aimed at guiding consumers toward more informed food choices and promoting healthier dietary behaviors (4). According to the Pan American Health Organization (PAHO), FOPNL represents a critical tool for supporting healthy eating and preventing noncommunicable diseases (NCDs) in the Americas (5). Among the different FOPNL formats, mandatory labeling has demonstrated greater effectiveness in conveying nutritional information to consumers. Its standardized design and prominent placement enhance visibility and comprehension, especially when compared to voluntary schemes such as Nutri-Score and the Health Star Rating system (6–9).

After 6 years of debate involving different social actors, Brazil approved a mandatory FOPNL regulation in 2020 (10–12). However, the resolution only came into effect in October 2022, with a phased implementation timeline based on product categories: October 8, 2023, for general food products on the market; October 8, 2024, for products from family farmers or rural entrepreneurs, economic solidarity organizations, individual small businesses, small-scale farmers, artisanal producers, and artisanal foods; and October 8, 2025, for non-alcoholic beverages in returnable packages (13). Due to industry interference, the initial implementation deadline was postponed from October 2023 to October 2024 through a Resolution that allowed them to deplete their stock of packages not yet compliant with the new rules (13, 14). However, following legal action filed in support of public health interests, the enforcement date was brought forward to April 15, 2024, after the Federal Court suspended the Resolution. Still, the other deadlines for compliance remained unchanged. The Brazilian regulation mandates the use of a magnifying glass symbol on the front of packages to indicate high levels of added sugar, saturated fat and sodium (Supplementary Figure 1). It also establishes specific requirements for the format, content, and legibility of the nutrition facts panel, and imposes restrictions on the use of nutrient content claims related to added sugar, saturated fat, or sodium on products displaying FOPNL for that nutrient (10, 11).

Public debate plays a crucial role in shaping the acceptance or rejection of public health policies. In 2024, the World Health Organization published a report emphasizing the importance of monitoring public attitudes toward health policies and the multiple ways through which such attitudes can be assessed. Public policy support can be understood as individuals’ attitudes toward the introduction, implementation, or continuation of a specific policy, ranging from strong opposition, through neutrality (with no strong feelings in either direction), to strong support (15). Such support can be measured using different methodological strategies, including large-scale quantitative surveys, which allow monitoring of trends over time, and qualitative approaches, such as interviews and focus groups, which provide insights into contextual factors and underlying reasons for these attitudes (15). Social media monitoring has also been used to capture public beliefs and perceptions of policies, although these results should not be interpreted as representative of the general population. Rather, they can offer valuable insights into the narratives, resistances, and factors associated with higher or lower levels of policy support (15).

Social media platforms have become significant channels for the dissemination of information, including scientific content, and for the expression of personal opinions. By broadening the scope of discussions, these platforms can influence public understanding and help shape social norms (16). Among digital platforms, X (formerly Twitter) stands out due to its dynamic format, which facilitates the real-time spread of opinions, news, and diverse content (17). As an open and public platform, X enables retrospective analyses and supports qualitative research on public engagement health-related (18). Recent studies have explored how X is used in health-related debates, highlighting both its potential as a tool for disseminating educational content and the challenges it poses due to the spread of misinformation. Ola et al. reported that the majority of health-related content on X is produced by the general public and is predominantly educational in nature, although its reliability varies (19). Similarly, Lynn et al. found that discussions around healthy eating are largely driven by non-specialist influencers and automated accounts, which can compromise information quality and generate misinformation (20).

Considering the context of FOPNL implementation in Brazil, the importance of monitoring public attitudes toward health policies and the growing relevance of social media as a communication channel, this study aimed to analyze how the Brazilian FOPNL policy has been represented and discussed on X.

This study addresses the following research questions: How do consumers discuss FOPNL from the moment of its approval until the end of the first compliance deadline? How do consumers position themselves in relation to this public policy over time?

Within this scenario, the present study seeks to advance the international literature by analyzing publicly available content on a social media platform, offering a replicable approach that can be applied to different countries as a means to assess public debate surrounding regulatory measures in the field of food and nutrition.

## 2 Materials and methods

### 2.1 Study design

This is a cross-sectional observational study based on posts published by personal user accounts on the social media platform X between October 2020 and April 2024.

### 2.2 Sampling

To extract posts from the platform X, automated tools were used. Scripts were developed to access publicly available content through the platform’s official Application Programming Interface (API), enabling systematic and automated data collection without the need for manual retrieval. The extraction targeted posts containing terms in Portuguese related to the FOPNL policy and its implementation, including: “Alto em açúcar adicionado” (“High in added sugar”), “Alto em gordura saturada” (“High in saturated fat”), “Alto em sódio” (“High in sodium”), “Rotulagem frontal” (“Front-of-package labeling”), “Rotulagem legislação” (“Legislation labeling”), “Rotulagem nutricional” (“Nutrition labeling”), “Legislação lupa” (“Legislation magnifying glass”) and “Rótulo alimento” (“Food label”). Variations in spelling, spacing, and the absence of diacritics were also considered. It should be noted however that using keywords could have limited the identification of other posts that were related to the topic but did not mention these words. Further, using a single social media platform could not capture the full range of perceptions among Brazilian consumers. However, despite the subjectivity present on social media, this study also has the strength of capturing the spontaneity and genuine opinions of users.

A total of 7,004 posts were extracted. Two eligibility criteria were applied: (1) the post had to include a direct reference to the Brazilian FOPNL policy, and (2) the content had to be written in Portuguese. After applying these criteria, 2,555 posts were considered valid. For this study, only posts from personal profiles, defined as accounts representing individual users, were included, resulting in a final sample of 2,323 posts (90.9%). These posts were compiled in an Excel spreadsheet containing the URL and date of publication to facilitate subsequent organization and analysis. All procedures followed ethical guidelines and complied with the platform’s terms of use.

### 2.3 Characterization of posts

To define the variables for analysis, 10% of the sample were classified according to: (i) publication category (science, informative, opinion, or regulation) (Supplementary Data 1), and (ii) positioning on FOPNL (positive, moderately positive, neutral, moderately negative, or negative) (Supplementary Data 2), as described by Irawan A et al. (2022) (21). An exploratory thematic content analysis was then conducted to characterize the message content, resulting in the identification of 26 themes: sugar, claims, Anvisa, criticism of the industry, criticism of the FOPNL, criticism of the FOPNL model, criticism of the product, disinterest due to FOPNL, consumer rights, non-sugar sweeteners, praise for the FOPNL, negative emotions, food choices, saturated fat, implementation, inquiry about the FOPNL, indifference to the FOPNL, industry, interest in products with FOPNL, improvement in labeling, nutrient profile, portion, regulatory process, reformulation, health risk and sodium (Supplementary Data 3).

Following this definition phase, the valid posts were individually and manually classified in the Excel spreadsheet according to publication category, positioning on FOPNL and theme. Coders underwent an initial training phase, which included a thorough review of the coding manual, detailed examples for each category, and group discussions to clarify ambiguous cases. Following this, coders completed practice sessions on a subset of posts to apply the criteria in a controlled setting, receiving feedback to enhance consistency. Regular calibration meetings were held throughout the coding process to discuss challenging cases, refine coding criteria, and ensure consistency across coders. One single post may have been defined in more than one theme. To ensure the reliability of the classification, inter-rater agreement was assessed using the prevalence-adjusted and bias-adjusted kappa coefficient (PABAK), with values above 0.60 considered satisfactory (22). All variables demonstrated substantial agreement, except for the neutral position, which showed moderate agreement (*k* = 0.44).

### 2.4 Data analysis

For analysis purposes, the 26 identified themes were grouped into eight broader thematic categories based on content similarity: (1) nutrients of concern, (2) health risk, (3) regulatory aspects, (4) no effect, (5) support for the regulation, (6) critiques and concerns, (7) decision-making, and (8) others. A single post could be assigned to more than one thematic category.

To ensure analytical consistency, 10% of the posts were jointly reviewed to discuss classification criteria. The remaining posts were independently analyzed. A thematic content analysis approach was used to describe and synthesize the qualitative data (23).

Descriptive statistics were used to assess the distribution of post frequency and thematic categories over the study period. In addition, 95% confidence intervals (CI) were calculated, and differences between groups were considered statistically significant when confidence intervals did not overlap. Non-exhaustive examples were selected to illustrate each category. To describe the relationship between thematic categories and positioning toward FOPNL, a diverging bar chart was constructed. Positive and moderately positive posts were grouped as favorable (plotted above the axis), while negative and moderately negative posts were grouped as unfavorable (plotted below the axis). Neutral posts were placed at the central axis. This visualization approach allowed for a clear depiction of response polarization and facilitated comparison across categories. The chart was created using Microsoft Excel, based on the relative frequencies of each category. All other analyses were conducted using Stata software, version 14.0.

## 3 Results

A total of 2,323 posts published in Portuguese on the social media platform X were analyzed. Based on the message content, the majority (89.97%) expressed personal opinions, followed by 11.11% classified as informative, 1.55% as related to regulation and only 0.09% as scientific content (data not shown in tables).

**Figure 1:**
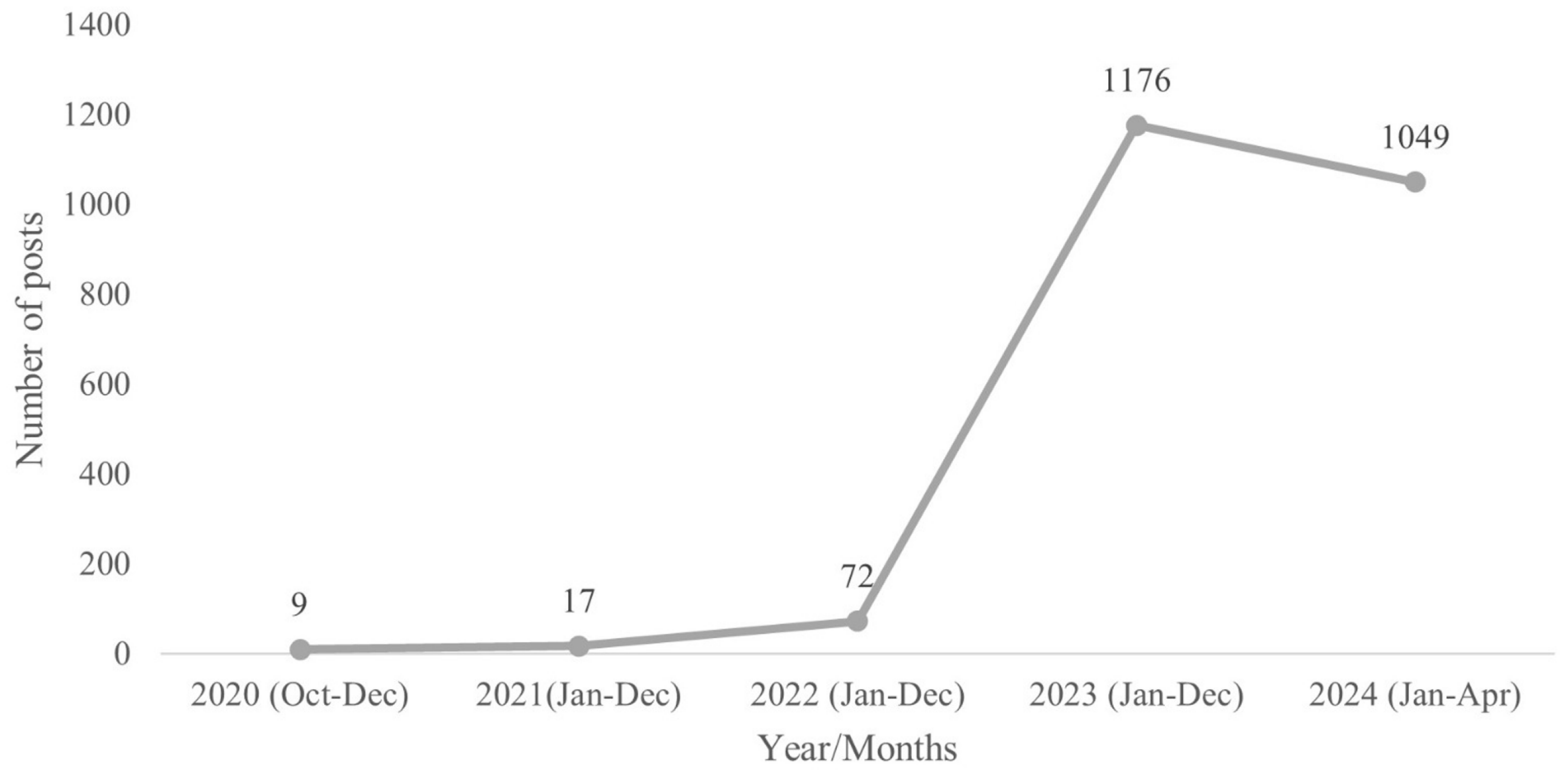
Time trend in personal posts on the social media platform X referring to front-of-package nutrition labeling (FOPNL) in Brazil, from October 2020 to April 2024.

The volume of posts showed an overall upward trend from 2020 to 2024, with distinct patterns over time. There was a slight increase in the early years: 9 posts were recorded in October 2020, 17 posts throughout 2021, and 72 in 2022. A substantial rise was observed in 2023, with a total of 1,176 posts, followed by a slight decrease in the first 4 months of 2024, during which 1,049 posts were recorded (Figure 1). Monthly trends are detailed in Supplementary Figure 2.

Regarding the content analysis, the most prevalent thematic category was “Nutrients of concern,” appearing in 92.04% of the posts. This was followed by “Critiques and concerns” (31.25%), “No effect” (29.83%) and “Decision-making” (26.47%). The remaining categories were less frequently observed, each accounting for less than 7% of the sample: “Regulatory aspects” (6.84%), “Support for the regulation” (6.20%), “Health risk” (3.06%) and “Others” (1.81%) (Table 1).

**Table 1:** Frequency of thematic categories related to front-of-package nutrition labeling (FOPNL) in personal posts on the social media platform X in Brazil, from October 2020 to April 2024.

Thematic categories and themes	*n*	%	95% CI
**Nutrients of concern**	**2,138**	**92.04**	**90.86–93.07**
Sugar	1,518	65.35	63.38–67.26
Saturated fat	1,098	47,27	45.24–49.30
Sodium	721	31,04	29.19–32.95
**Critiques and concerns**	**726**	**31.25**	**29.40–33.17**
Criticism of the product	217	9.34	8.22–10.59
Negative emotions	215	9.26	8.14–10.50
Criticism of the FOPNL	195	8.39	7.33–9.59
Inquiry about the FOPNL	94	4.05	3.32–4.93
Criticism of the industry	67	2.88	2.27–3.65
Criticism of the FOPNL model	39	1.68	1.23–2.29
**No effect**	**693**	**29.83**	**28.00–31.73**
Indifference to the FOPNL	687	29.57	27.75–31.46
Interest in products with FOPNL	464	19.97	18.40–21.65
**Decision-making**	**615**	**26.47**	**24.72–28.31**
Disinterest due to FOPNL	493	21.22	19.61–22.93
Food choices	349	15.02	13.63–16.54
**Regulatory aspects**	**159**	**6.84**	**5.88–7.95**
Anvisa	47	2.02	1.52–2.68
Implementation	62	2.67	2.08–3.41
Reformulation	34	1.46	1.05–2.04
Regulatory process	19	0.82	0.52–1.28
Nutrient profile	15	0.65	0.39–1.07
Consumer rights	9	0.39	0.20–0.74
Non-sugar sweeteners	2	0.09	0.02–0.34
**Support for the regulation**	**144**	**6.20**	**5.29–7.26**
Improvement in labeling	108	4.65	3.86–5.58
Praise for the FOPNL	81	3.49	2.81–4.31
**Health risk**	**71**	**3.06**	**2.43–3.84**
**Others (< 1%)**	**42**	**1.81**	**1.34–2.44**
Industry	20	0.86	0.55–1.33
Claims	16	0.69	0.42–1.12
Portion	7	0.30	0.14–0.63

FOPNL, Front-of-package nutrition labeling; Anvisa, The Brazilian National Health Surveillance Agency; 95% CI, 95% confidence interval. Bold values represent the names of thematic categories obtained after grouping similar themes.

The frequency of thematic categories over time revealed distinct distribution patterns. The categories *“Nutrients of concern,” “No effect,”* and *“Others”* were more prominent only in the years 2023 and 2024, appearing in 94.47 and 93.71%, 30.53 and 31.46%, and 1.62 and 0.86% of posts, respectively. Conversely, the category *“Regulatory aspects”* was more frequent in the earlier years of the analysis, accounting for 66.67% of posts in 2020, 41.18% in 2021, and 61.11% in 2022. Similarly, *“Support for the regulation”* reached higher frequencies in 2021 (35.26%) and 2022 (23.61%), before declining in subsequent years. The remaining categories showed relatively stable frequencies across the study period (Table 2).

**Table 2:** Evolution of thematic categories in personal posts related to front-of-package nutrition labeling (FOPNL) on the social media platform X in Brazil, from October 2020 to April 2024.

Thematic categories	*n*	2020			2021		*n*	2022		*n*	2023		*n*	2024	
		%	95% CI	*n*	%	95% CI		%	95% CI		%	95% CI		%	95% CI
Nutrients of concern	1	11.11	1.36–53.16	5	29.41	12.44–55.00	38	52.78	41.21–64.05	1,111	94.47	93.01–95.64	983	93.71	92.07–95.03
Health risk	0	0.00	-	0	0.00	-	4	5.56	2.08–13.98	46	3.91	2.94–5.18	21	2.00	1.31–3.05
Regulatory aspects	6	66.67	31.48–89.69	7	41.18	20.54–65.46	44	61.11	49.36–71.69	45	3.83	2.87–5.09	57	5.43	4.21–6.98
No effect	0	0.00	-	0	0.00	-	4	5.56	2.08–13.98	359	30.53	27.98–33.22	330	31.46	28.71–34.34
Support for the regulation	1	11.11	1.36–53.16	6	35.29	16.35–60.34	17	23.61	15.16–34.84	61	5.19	4.05–6.61	59	5.62	4.38–7.19
Critiques and concerns	3	33.33	10.30–68.52	6	35.29	16.35–60.34	15	20.83	12.92–31.82	394	33.50	30.86–36.25	308	29.36	26.68–32.19
Decision-making	0	0.00	-	3	17.65	5.59–43.67	16	22.22	14.03–33.34	373	31.72	29.12–34.44	223	21.26	18.88–23.84
Others	3	33.33	10.30–68.52	2	11.76	2.83–37.91	9	12.50	6.60–22.40	19	1.62	1.03–2.52	9	0.86	0.45–1.64

95% CI, 95% confidence interval.

With respect to positioning on FOPNL, the majority of posts (67.59%) exhibited a neutral position. A smaller proportion expressed favorable views, with 15.24% showing positive perceptions (9.04% moderately positive; 6.20% positive), while 17.18% reflected unfavorable views (13.69% moderately negative; 3.49% negative) (data not shown in tables).

**Figure 2:**
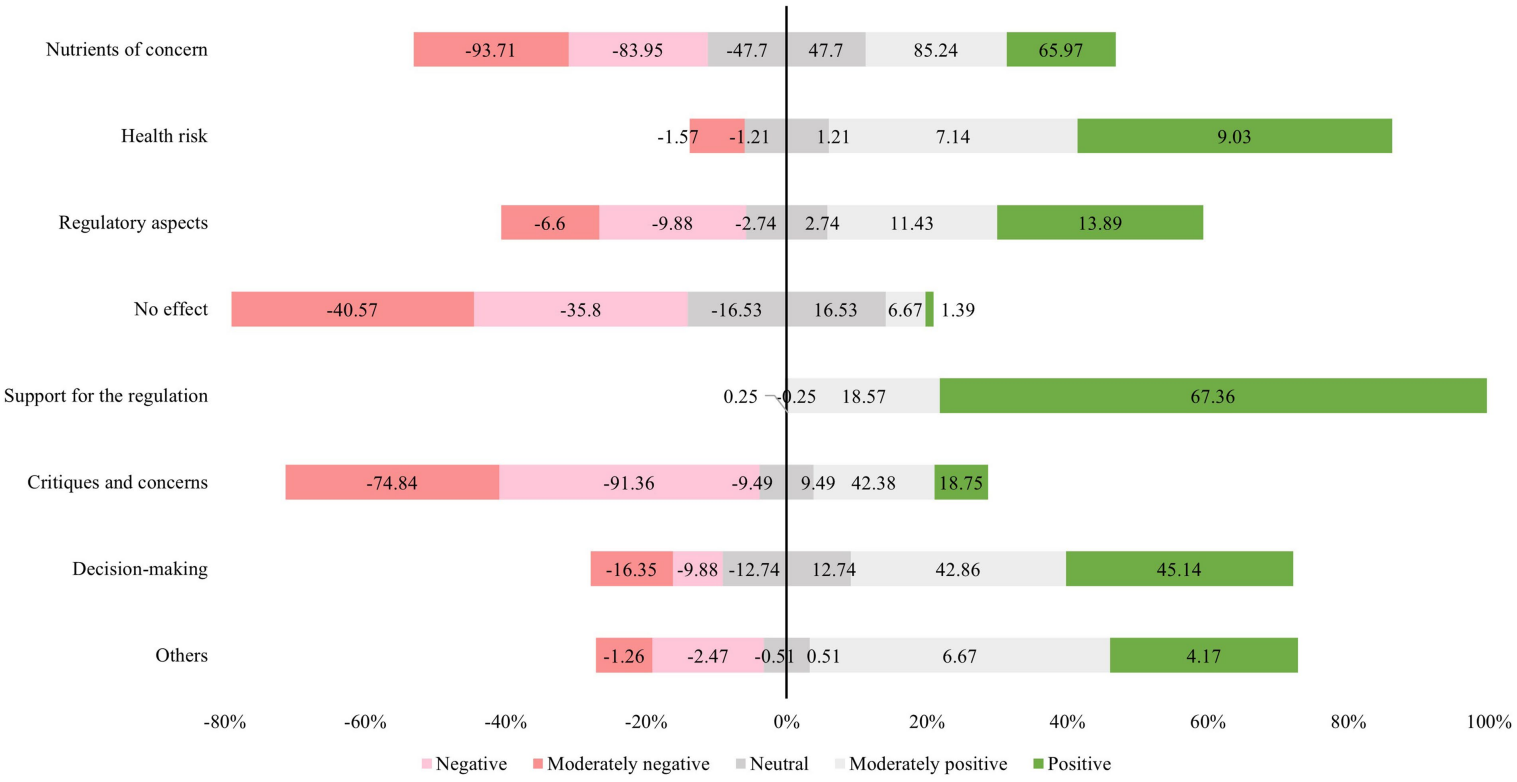
Distribution of the positions of posts related to front-of-package nutrition labeling (FOPNL) on the social media platform X in Brazil, stratified by thematic category, from October 2020 to April 2024. The position was classified as positive, moderately positive, neutral, moderately negative, or negative.

Analysis of positioning within each thematic category revealed a predominance of positive perceptions in certain areas. In the “Support for the regulation” category, the vast majority of posts were classified as either moderately positive (18.57%) or positive (67.36%). Similarly, the “Decision-making” category showed high levels of favorable attitudes (42.86% moderately positive; 45.14% positive), followed by “Regulatory aspects” (11.43% moderately positive; 13.89% positive) and “Health risk” (7.14% moderately positive; 9.03% positive). In contrast, negative perceptions were more prevalent in the “Critiques and concerns” category (78.84% moderately negative; 91.36% negative) and “No effect” (40.57% moderately negative; 35.80% negative), highlighting a predominance of unfavorable sentiment toward the regulation in these themes. The “Nutrients of concern” category presented a more balanced distribution of perceptions, with both positive (85.24% moderately positive; 65.97% positive) and negative (93.71% moderately negative; 83.95% negative) evaluations being highly represented, indicating a high level of engagement, both supportive and critical. The “Others” category showed a lower frequency of evaluative tone, with 10.84% of posts classified as positive (6.67% moderately positive; 4.17% positive) and a smaller proportion expressing negative sentiment (2.47% moderately negative; 1.26% negative) (Figure 2).

The thematic categories are described below, along with illustrative examples that capture their defining characteristics. These examples are not exhaustive and were selected based on the clarity and prominence of the tone expressed toward the regulation.

### 3.1 Nutrients of concern

Nutrients of concern are central and connected to all the thematic categories, as they correspond to the nutrients highlighted in Brazilian FOPNL (added sugar, saturated fat and sodium). References to critical nutrients ranged from technical descriptions to expressions of discomfort, irony or humor. Despite the thematic diversity, many posts did not convey an explicit value judgment, merely reporting the presence of a “high in” label, thereby masking the author’s stance. Among the three nutrients highlighted, added sugar stood out as the most frequently cited.

“I’m really enjoying these new labels on food packages—“high in added sugar,” “high in saturated fat,” “high in sodium. Super clear and easy to see, and they show up on things you would not even expect. It really helps you make better choices when shopping.” (Post published on June 11, 2023).

“Someone online said they stopped eating foods with the “high in sugar, sodium, and fat” label. Have you done the same or do you not really care?” (Post published on October 17, 2023).

I was eating an amazing chocolate from [brand] when I noticed a label on the package that said “high in saturated fat, added sugar and sodium. Like I care about that, if I wanted to be healthy, I’d be eating fruit.” (Post published on June 11, 2023).

### 3.2 Critiques and concerns

The messages within this thematic category encompassed three main aspects: the FOPNL model, the products displaying front-of-package nutrition labels and the reactions elicited by the presence of such labels, including doubt, questioning, and criticism. A common thread across these aspects was the expression of dissatisfaction or discomfort regarding FOPNL. The most prevalent themes were criticism directed at specific products and the expression of negative emotions. In the initial years of the analysis, users frequently questioned the effectiveness and clarity of the labeling system. Additionally, skepticism regarding industry practices emerged, with some posts suggesting that companies might adopt strategies to offset or downplay the impact of the warning labels. As time progressed, concerns shifted toward the nutritional composition of labeled products and a range of emotional reactions to the labels was observed. Some users reported feelings of guilt and indicated that FOPNL could provoke adverse emotional responses related to food, using terms such as “anxious,” “terrified,” “guilty,” and expressions like “the magnifying glass annoys me” to describe their experience.

“Who decided to label everything I eat as ‘high in added sugar’? Now I’m left with a guilty conscience.” (Post published on November 8, 2023).

### 3.3 No effect

In posts related to this theme, users frequently reported that the presence of FOPNL did not change their perception of the product or reduce their intention to consume it. Posts with ironic or sarcastic undertones were common, indicating that FOPNL was not only ineffective in discouraging consumption but, in some cases, appeared to reinforce interest in the labeled products. This reaction was particularly observed in references to items high in added sugar and saturated fat, where the magnifying glass symbol was perceived by some users as attractive rather than cautionary. Additionally, several users associated FOPNL-labeled products with emotional eating, especially in situations involving reward, frustration, or happiness. In these cases, the hedonic value or emotional relief derived from food consumption seemed to outweigh the impact of nutritional warnings.

“And by the way, it is no surprise that a chocolate bar is high in sugar and saturated fat. It makes no difference to me.” (Post published on March 9, 2024).

### 3.4 Decision-making

At the beginning of the analysis period, many users expressed uncertainty regarding the effectiveness of FOPNL in influencing food choices. As the study period progressed, some posts suggested that the presence of FOPNL affected purchasing decisions, with reports of disinterest or even rejection of labeled products. In particular, users were often surprised to see FOPNL on products that carried health-related claims. In such cases, the label was perceived as a tool that reduced the likelihood of being misled by marketing, enhancing the sense of transparency in food labeling.

“The label stating ‘high in fat and added sugar’ was highly effective in my case—I returned the product to the shelf and searched for an alternative without the label” (Post published on December 7, 2023).

### 3.5 Regulatory aspects

The greater prominence of this theme at the beginning of the study period is attributable to the predominantly technical, informative and inquisitive nature of the posts. Key concerns raised in this context included: (1) the perceived influence of the food industry in promoting a FOPNL model that does not align with the best available scientific evidence; (2) criticism that the design adopted in Brazil does not constitute a true warning label; and (3) concerns that the threshold values for triggering FOPNL display are too lenient. These discussions were often linked to broader claims about consumers’ rights to clear and adequate nutritional information. As the discourse evolved throughout the study period, the tone of the posts shifted. Praise for the National Health Surveillance Agency (Anvisa) for approving the regulation became more frequent, although some ironic commentary on the implementation of FOPNL persisted.

“I admit that the new front-of-package label regulation for food, approved by Anvisa, is a win for consumers. The food industry did not like this and will have to inform what it has always hidden: high levels of added sugar, salt, and fats” (Post published on October 04, 2022).

### 3.6 Support for the regulation

Posts within this thematic category emphasized perceived benefits associated with FOPNL. This labeling system was frequently acknowledged as a mechanism for enhancing consumers’ capacity to make informed dietary choices, primarily through increased clarity and transparency of nutritional information. Notably, several posts underscored the role of FOPNL in exposing the actual nutritional composition of products marketed with health-related claims, particularly those found to contain elevated levels of nutrients of concern.

“This is excellent for foods and beverages whose labels claim that they are natural and healthy, but when this label is added everyone finds out about the deception.” (Post published on March 15, 2024).

### 3.7 Health risk

Posts within this thematic category addressed potential health risks associated with the consumption of products displaying FOPNL. Several users explicitly referenced specifics NCDs, acknowledging the role that nutrients of concern may play in them. These references were framed both from the standpoint of disease prevention and in the context of managing pre-existing health conditions.

“Look at this, @user and @user2. They indicate that the chocolate egg is not only high in sugar, which is seen as a warning to diabetics, but also high in saturated fat, which might make people sick.” (Post published on April 5, 2023).

### 3.8 Others

Themes that appeared with a frequency of less than 1% were consolidated under this category. These posts addressed a range of isolated or less prevalent issues, including dissatisfaction with specific brands, perceived contradictions between the FOPNL and other nutritional or marketing claims on the packaging, and the strategic use of serving size or net weight to emphasize or obscure certain nutritional information.

“Such a fine thing to do, @(brand) | @(supermarket). Hiding information on high sodium content from consumers? All trays at the point of sale look the same way. @(consumer protection agency). This is absurd” (Post published on February 3, 2024).

## 4 Discussion

One approach to assessing the degree of societal engagement with regulatory measures is through the analysis of user-generated content over time. The present findings demonstrate a progressive increase in the volume of posts throughout the study period, which appears to reflect growing public discourse surrounding the implementation of FOPNL in Brazil. Notably, the surge in posts observed between 2023 and 2024 coincided with key regulatory milestones, particularly the initial compliance deadlines for products already on the market and for new product formulations, which occurred between October 2023 and April 2024 (10, 13, 24).

As consumers began to encounter FOPNL on processed and UPFP, this visibility appeared to catalyze renewed public debate and expressions of opinion, particularly on social media platforms. A key event that may account for the spike in posts during this period was Easter 2023. During this time, all new products or those with reformulated compositions were required to comply with the updated labeling regulation. As a result, traditional Easter items such as chocolate eggs began to display FOPNL indicating “high in added sugar” and “high in saturated fat” (25, 26). Additionally, the mobilization of civil society organizations, as well as media coverage and increased visibility in mass media, likely contributed to encouraging individual expression and engagement. Collectively, these milestones appear to have fostered heightened public interest and involvement, leading to a broader dissemination of information, opinions and attitudes regarding the regulation.

The predominance of opinion-based posts suggests that the material analyzed primarily reflects individual perceptions of FOPNL, thereby emphasizing the centrality of consumers’ personal experiences and interpretations in shaping their responses to the regulatory measure. This dimension is essential for assessing the extent of social acceptance or resistance to the policy. Monitoring social media can offer insights into the drivers of public support or opposition about policies (15). Regulatory initiatives that are perceived as congruent with the values and expectations of the population are more likely to be regarded as legitimate, which can, in turn, enhance public trust in institutions and promote adherence to the established guidelines (27).

Among the thematic categories, references to nutrients of concern were the most prevalent. This topic appeared consistently across the database and was strongly linked with almost all the other categories, with diverse content reflecting the central role of these nutrients highlighted in Brazilian FOPNL. These mentions were associated with both favorable and unfavorable evaluations of the regulation and also appeared in posts conveying factual information, posing questions or reflecting varying degrees of influence on purchasing decisions. Prior to the implementation of the Brazilian FOPNL policy, experimental evidence had already demonstrated the potential of FOPNL to support informed and promote healthier food choices among Brazilian consumers (28). Furthermore, a recent study examining the influence of three label elements (FOPNL, brand and nutritional claims) on perceived product healthiness found that FOPNL significantly reduced healthfulness perceptions, whereas unregulated elements such as nutrition claims had the opposite effect (29). These findings align with the qualitative results of the present study, as many posts reported that the presence of FOPNL enhanced informational clarity and shaped health perceptions, particularly in relation to products that simultaneously carried health-oriented marketing advertising elements.

The temporal evolution of thematic categories reveals important shifts in the nature of public discourse. Although regulatory aspects and support for the regulation were more prominent in the initial years of the study, their frequency declined over time, while discussions around nutrients of concern, no effect, and others became predominant in 2023 and 2024. This shift suggests that early debates were primarily driven by technical arguments and regulatory procedures, often voiced by actors directly engaged in the policy-making process. In contrast, more recent discussions appear less technical and more closely connected to consumer experiences, everyday practices, and subjective perceptions of the policy’s impact. These findings indicate a shift in the nature of public discourse over time and reveal heterogeneous consumer responses to the regulation. The coexistence of convergent and divergent perspectives highlights the complex social reception of the FOPNL policy and suggests that its implementation may yield varied behavioral effects across different segments of the population.

The analysis of consumer perceptions expressed on the social media platform X offered nuanced insights into not only the predominant themes, but also the meanings ascribed to them by users. This type of qualitative exploration is critical for capturing the broader contours of the public debate and for identifying areas of support, resistance, and ambivalence regarding the implementation of the regulatory measure (30). The findings revealed the existence of favorable perceptions, particularly in relation to endorsement of the policy, the influence of FOPNL on purchasing behavior, and discussions surrounding regulatory processes. In contrast, negative perceptions were generally characterized by skepticism, criticism, and expressions of disengagement with the potential impact of the labeling. Notably, the theme of nutrients of concern exhibited a more heterogeneous profile, encompassing both supportive and oppositional viewpoints, which suggests a complex and multifaceted public reception of this regulatory initiative. Another important point to note is the surprise reactions when finding products with FOPNL on labels with health-related claims, for example. These reactions may happen because health-related claims can mislead consumers about the nutritional composition of the product and can have an effect on purchase decisions (31).

Most posts were classified as neutral, which may have been due to the challenge in understanding the exact tone used by users, often with irony and sarcasm, as in the thematic categories of ‘no effect’ and ‘regulatory aspects’, but there were also posts in which the tone used was more objective, such as in informative posts. It is worth noting that the characteristics of social media itself can both hinder and help in identifying feelings, such as the use of short messages and emoticons (32). Although this result may appear less informative than explicit expressions of support or opposition, it provides important insights into the dynamics of public debate. Public support for policies should be understood as a spectrum ranging from strong opposition to strong support, with neutrality as an inherent and meaningful category (15). The predominance of neutral posts suggests that a large share of interactions was centered on circulating information, reporting events, or expressing uncertainty, rather than reflecting strong attitudes. This finding highlights that the debate on FOPNL during the analyzed period was not fully polarized, leaving room for opinion formation and potential shifts as new evidence or arguments emerge.

The use of social media as a platform for expressing opinions on public food and nutrition policies has been explored in studies conducted in various international contexts. For example, a study carried out within the European Union analyzed posts on platform X regarding different FOPNL systems across the region, examining content, sentiment, and network dynamics (20). Similarly, research conducted in the United Kingdom investigated public perceptions and awareness of mandatory calorie labeling on food and beverage items sold by large out-of-home food outlets, based on user responses to posts on X (33). Both studies revealed a coexistence of supportive and critical viewpoints, consistent with the findings of the present study. Overall, negative perceptions and skepticism regarding the efficacy of such policies in influencing dietary behavior and improving health outcomes were predominant. Notably, in the UK context, concerns were frequently raised about the potential adverse effects of calorie labeling policies on individuals with eating disorders (33). Although the regulatory frameworks differ from the FOPNL policy implemented in Brazil, the present analysis similarly identified user-generated content expressing concern that FOPNL could elicit feelings of guilt and potentially exacerbate disordered eating behaviors among individuals with a history of eating disorders.

Notably, the positive perceptions expressed by consumers suggest that FOPNL is being recognized as an effective public health strategy. These findings highlight key dimensions of the social acceptability of the policy, particularly among individuals who acknowledged its potential to address nutrition-related health challenges. Posts that explicitly endorsed the regulation and emphasized its relevance for population health, as well as those that indicated a direct influence of FOPNL on consumer behavior, were especially prominent.

The considerable volume of user-generated content related to FOPNL on social media platform X, along with the substance of these messages, suggests heightened consumer awareness and attentiveness in food purchasing decisions, even when such decisions involve products bearing FOPNL. Overall, the diversity of consumer responses underscores that the provision of clear and accessible nutritional information on labels can meaningfully influence food choice, reinforcing that FOPNL systems can be supported by the public, especially when they offer an easy-to-understand design and information (34).

### 4.1 Limitations and strengths

This study presents limitations. The exclusive focus on a single social media platform may not capture the full range of perceptions among Brazilian consumers, particularly those who do not engage in online discourse or prefer not to share their views in digital environments. Additionally, the use of predefined keywords may have limited the identification of relevant posts that addressed the topic without employing the selected terms. Given the dual formal and informal nature of social media platforms, the content analyzed is inherently subjective, which may have influenced the interpretation of the posts.

Despite these limitations, a key strength of this study lies in its analysis of spontaneously generated content by consumers from across Brazil, offering a unique opportunity to examine public perceptions on a national scale, despite the country’s vast geographical and cultural diversity. Importantly, the organic nature of social media discourse allows for the expression of genuine consumer opinions, which are not shaped by the expectations or presence of researchers, thereby contributing to a more authentic understanding of public sentiment regarding FOPNL.

## 5 Conclusion

The implementation of the FOPNL policy in Brazil elicited a wide range of consumer perceptions, reflecting the multifaceted social reception of the measure. Public health policies such as FOPNL are instrumental not only in fostering healthier food environments, but also in shaping social norms related to food choices and nutritional awareness. The findings of this study revealed a progressive increase in consumer engagement over time on X, suggesting that the regulation has become a topic of growing public interest and relevance. It was also possible to understand consumers’ position regarding the public policy by analyzing attitudes and sentiments related to FOPNL through the social media posts. This upward trend in posts, combined with the diversity of meanings attributed to the FOPNL, provides insight into the broader social and behavioral effects of the policy. In a context marked by the high prevalence of NCDs, such regulatory measures represent strategies to increase the visibility of nutrients of concern and to support consumers in making more informed food choices. These results underscore the importance of monitoring not only the implementation and technical effectiveness of public health regulations, but also the ways in which consumers perceive and respond to them. Incorporating the monitoring of consumer perceptions into policy evaluation processes is therefore essential for enhancing regulatory legitimacy, strengthening public trust, and informing the continued development of nutrition labeling initiatives, such as FOPNL in Brazil and in other countries around the world with similar regulatory measures. Future research should explore additional social media platforms to enlarge the coverage of public debate surrounding regulatory measures in the field of food and nutrition, while also incorporating demographic data collection when available to improve representativeness of the sample.

## Data Availability

The original contributions presented in the study are included in the article/Supplementary material, further inquiries can be directed to the corresponding author.
