# Supplementary material for: Brazilian front-of-package nutrition labeling: consumer perceptions on social media platform X
Source: Front Nutr. 2025 Oct 21;12:1666794. doi: 10.3389/fnut.2025.1666794 (PMC12583025; doi:10.3389/fnut.2025.1666794)
Supplement: Supplementary file 1 [file Table_1.docx]

Supplementary Material

# Supplementary Data

**Supplementary Data 1** - Publication categories: definitions, inclusion and exclusion criteria

| **Categories** | **Definition** | **Inclusion criteria** | **Exclusion criteria** |
| --- | --- | --- | --- |
| **Science** | If the tweet is related or is based on scientific evidence, code 1. If it is irrelevant to this category, code 0. | Consider it even without scientific reference to the evidence. | Do not consider it if the scientific basis is not clear |
| **Information** | If the tweet is a statement or claim about FOPNL, or is related to information available of courses/materials about FOPNL, code 1. If it is irrelevant to this category, code 0. | Consider posts that contain only facts about FOPNL, without personal content.  Consider posts that share information on courses about FOPNL or other types of business information. | n/a |
| **Opinion** | If the person expresses an opinion about food labeling systems, a sentiment, a view, or makes a judgment unrelated to scientific knowledge or a scientific fact, code 1. If it is irrelevant to this category, code 0. | It includes posts that present some personal perception or experience about FOPNL, whether in the form of praise, criticism, or irony.  **It may include questions or inquiries out of curiosity, or attempts to obtain information.* | n/a |
| **Regulation** | If the tweet is related to the regulatory process, code 1. If it is irrelevant to this category, code 0. | Consider publications that share information about: technical content about the rule; implementation deadlines; product reformulation. | Mention of Anvisa without linking it to the regulatory process |

**Supplementary Data 2** - Posts positions to the front-of-package nutrition regulation

| **Position** | **Definition** |
| --- | --- |
| Positive | If the tweet is clearly praising/advocating Anvisa’s FOPNL, code 1. If it is irrelevant to this category, code 0. |
| Moderately positive | If the tweet is partially praising/advocating Anvisa’s FOPNL, code 1. If it is irrelevant to this category, code 0. |
| Neutral | If one cannot identify whether the tweet is clearly or partially in favor of or against Anvisa’s FOPNL, code 1. If it is irrelevant to this category, code 0. |
| Moderately negative | If the tweet is partially criticizing Anvisa’s FOPNL, code 1. If it is irrelevant to this category, code 0. |
| Negative | If the tweet is clearly criticizing Anvisa’s FOPNL, code 1. If it is irrelevant to this category, code 0. |

**Supplementary Data 3** - Descriptions and analytical justifications of identified themes in posts

| **Themes** | **Description** | **Justification** |
| --- | --- | --- |
| Sugar | When there is mention of the high magnifying glass for added sugar/sugar or any mention of this nutrient | Nutrient present in the Brazilian FOPNL |
| Claims | Mention of health claims, nutrition, nutrients, and other. For example: amount and percentage of specific ingredients/nutrients, claims of healthiness, etc. | Possible presence of claims on labels and their influence on product perception |
| Anvisa | When there is mention of Anvisa | Agency that regulates FOPNL in Brazil |
| Criticism of the industry | Criticism of the food industry | Manufacturing sector that is involved in the process of implementing the rule |
| Criticism of FOPNL | Direct criticism of FOPNL | To identify criticism of the Brazilian FOPNL |
| Criticism of the FOPNL model | Criticism of the model /design chosen for FOPNL | To identify criticism of the Brazilian FOPNL model |
| Criticism of the product | Criticism of the product/food as a whole | To identify criticism of products and their relationship with FOPNL in Brazil |
| Disinterest due toFOPNL | The fact that the product contains FOPNL discourages consumers to eat/purchase the product | To check if the presence of Brazilian FOPNL negatively impacts one’s interest in the product |
| Consumer rights | Mentions information about consumer rights | Brazil has a Consumer Protection Code, and information on labeling must be in accordance with this code |
| Non-sugar sweetener | Mention of sweeteners | Probable partial or total replacement of sugars with sweeteners owing to the FOPNL for added sugars, based on the Chilean experience |
| Praise for the FOPNL | Praise or positive comments about FOPNL | To recognize compliments to the Brazilian FOPNL |
| Negative emotions | When there is some negative emotion (sadness, fear of consumption, fear, frustration, guilt, etc.) owing to the presence of FOPNL on the product | To identify if the presence of Brazilian FOPNL arouses negative emotions |
| Food choices | When the tweet makes it clear that the person has made a food choice using FOPNL for their purchase or consumption decision | To identify the influence of FOPNL on food choices |
| Saturated fat | When there is mention of FOPNL for high fat content or any mention of this nutrient | Nutrient present in FOPNL in Brazil |
| Implementation | When there is mention about the process for implementation of FOPNL | To identify mentions of the implementation of the FOPNL rule |
| Inquiry about the FOPNL | Any inquiry or question about FOPNL | To identify inquiries induced by FOPNL in Brazil |
| Indifference to the FOPNL | When, despite the presence of FOPNL, an individual is not impacted by it and does not change their opinion about the product - it includes a tone of irony  **Not all tweets that were identified with indifference, will necessarily be identified as an interest in products with FOPNL, but the opposite is true.* | To identify if the presence of FOPNL in Brazil does not impact one’s interest in the product |
| Industry | Mention of the food industry | Productive sector that is involved in the process of implementing the FOPNL rule |
| Interest in products with FOPNL | Despite the presence of FOPNL, the interest in consuming the product remains or increases  **The tweets that are identified as attracting one’s interest will also be accompanied by indifference to FOPNL.* | To identify whether the presence of Brazilian FOPNL increases one’s interest in the product |
| Improved in labeling | It mentions the improvement in food labeling with FOPNL and a better understanding of the nutritional composition of products | Understanding FOPNL as an advance for access to information |
| Nutrient profile | It mentions the nutrient profile used in FOPNL | The FOPNL rule contains a cut-off point for each of its nutrients that must be declared on the front-of-package information label of eligible products |
| Portion | Mentions of diving food into portions | FOPNL considers 100 g/ml of the products for the warning statement on the label, but one can also identify the amount of each of these nutrients per serving |
| Regulatory process | It mentions information about the regulatory process for FOPNL | To identify mentions of the regulatory process for FOPNL |
| Reformulation | It mentions reformulation of the product | Products may be reformulated in order not to include FOPNL |
| Health risk | It mentions possible health risks when consuming products with FOPNL or specifically mentions some chronic non-communicable disease (e.g. diabetes, high blood pressure/arterial hypertension, high cholesterol, overweight/obesity, cancer, etc.) | The Brazilian FOPNL model highlights three nutrients whose excessive consumption is associated with health risks and the development of chronic non-communicable diseases |
| Sodium | When there is mention of FOPNL for high sodium or any mention of this nutrient | Nutrient present in FOPNL in Brazil |

# Supplementary Figures and Tables

## Supplementary Figures

**Supplementary Figure 1.** Brazilian front-of-package nutrition labeling model


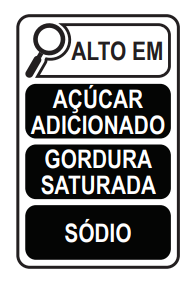


**Supplementary Figure 2.** Monthly increase in user-generated posts on Front-of-Package Nutrition Labeling in Brazil on Platform X, from October 2020 to April 2024

| **(A) Monthly trend - 2020** |
| --- |
|  |
| **(B) Monthly trend - 2021** |
|  |
| **(C) Monthly trend - 2022** |
|  |
| **(D) Monthly trend - 2023** |
|  |
| **(E) Monthly trend - 2024** |
|  |
